# Supplementary material for: Impact of exposure frequency on disease burden of the common cold – A mathematical modeling perspective
Source: PLoS One. 2025 Oct 22;20(10):e0334527. doi: 10.1371/journal.pone.0334527 (PMC12543168; doi:10.1371/journal.pone.0334527)
Supplement: S2 File — This file contains the source code needed to reproduce all calculations carried out in the entire manuscript. (HTML) [file pone.0334527.s002.html]

Source code of article “Impact of exposure frequency on disease burden of the common cold - a mathematical modeling perspective”


# Source code of article “Impact of exposure frequency on disease burden of the common cold - a mathematical modeling perspective”

```
# Set random seed:
set.seed(1)

# Load required libraries:
library('tidyverse') 
library('patchwork')
library('deSolve')
library('latex2exp')

# Set the ggplot theme and plot colors
theme_set(theme_bw())
cbPalette <- c("#E69F00", "#0072B2", "#CC79A7", "#009E73", "#D55E00", "#56B4E9")
```

```
# Create and plot data for exemplary virus strains
x_max <- 20
(fig_1 <- tribble(~ x_left, ~ x_right, ~ y_bottom, ~ y_top, ~ group,
        0, 0.2, 0.6, 0.7, 'Infection with Virus "X.1"',
        0.25, 0.3, 0.6, 0.7, 'Infection with Virus "X.1"',
        0, 0.2, 0.45, 0.55, 'Infection with Virus "X.1"',
        0.75, 0.95, 0.45, 0.55, 'Infection with Virus "X.1"',
        0, 0.2, 0.3, 0.4, 'Infection with Virus "X.1"',
        0.25, 0.37, 0.3, 0.4, 'Infection with Virus "X.2"') |> 
  mutate(x_left = x_left * x_max,
         x_right = x_right * x_max) |> 
  ggplot(aes(xmin = x_left, 
             xmax = x_right, 
             ymin = y_bottom, 
             ymax = y_top, 
             fill = group)) + 
  xlim(0, x_max) + 
  labs(x = 'Time', fill = '') +
  scale_y_continuous(labels = str_wrap(
    c('Scenario C: Early reinfection with cross-reactivity', 
      'Scenario B: Long time between infections', 
      'Scenario A: Early reinfection'), 25), 
    breaks = c(0.35, 0.5, 0.65)) + 
  geom_rect() +   
  theme(
    axis.text.x = element_blank(),
    axis.ticks.x = element_blank(),
    panel.grid.major = element_blank(), 
    panel.grid.minor = element_blank(),
    axis.line.x = element_line(
      arrow = arrow(type='closed', length = unit(10, 'pt')))
  ) +
  scale_fill_manual(values = cbPalette))
```

```
# Transition probability function from state I to S
transition_prob_I_to_S <- function(theta, a, d, c) {
  a * exp(-d * theta) + c
}

# Set parameters for the scenarios:
scenario_params <- tribble(~ scenario_name, ~ a, ~ d, ~ c, 
        'No specific immunity', 0, 0, 0.02,
        'Medium specific immunity', 0.49, 1 / 10, 0.015,
        'Strong specific immunity', 0.99, 1 / 20, 0.01)

# Plot the transition probability function for some parameter configurations
(fig_2 <-  scenario_params |> 
  mutate(scenario_name = fct_inorder(scenario_name),
         params = paste0('a = ', a, 
                         '\nd = ', d,
                         '\nc = ', c)) |> 
  expand_grid(theta = 0:100 * 10) |> 
  mutate(value = transition_prob_I_to_S(theta, a, d, c)) |> 
  ggplot(aes(theta, value, colour = scenario_name)) + 
  labs(x = expression(paste('Time since last recovery (', theta[i], ')')), 
       y = TeX('Transition probability $p_{I \\rightarrow S}$'),
       colour = 'Scenario') +
  geom_line() + 
  geom_label(aes(x = 500, y = 0.85, label = params, hjust = 0), 
             inherit.aes = FALSE) +
  facet_wrap(vars(scenario_name)) +
  scale_color_manual(values = cbPalette) + 
  theme(legend.position = 'none'))
```

```
# Set ODE object
sir_ode_immunity <- function(t, state, pars) {
  with(as.list(c(state, pars)), {
    dS <- - beta_1 * beta_2 * I * S + delta * R
    dI <- beta_1 * beta_2 * I * S - gamma * I - alpha * beta_2 * I
    dR <-  gamma * I + alpha * beta_2 * I - delta * R
    return(list(c(dS = dS, dI = dI, dR = dR)))
  })
}

# Restructure ODE object
df_deSolve_result <- function(result) {
  tib <- as_tibble(unclass(result))  
  vars <- colnames(result)[-1]
  long <- pivot_longer(tib, cols = all_of(vars))
  long
}

# Calculate ODE solution
make_df_sir_solution <- function(beta_1 = 0.7, beta_2 = 0.7, gamma = 0.1, delta = 0.1, alpha = 0.5) {
  infected_initial <- 0.01
  initial_condition <- c(S = 1 - infected_initial, I = infected_initial, R = 0)

  # Generate ODE
  timepoints <- seq(0, 50, by = 0.1)
  ode(y = initial_condition,
      times = timepoints,
      func = sir_ode_immunity,
      # Set parameter values
      parms = list(alpha = alpha,
                   beta_1 = beta_1,
                   beta_2 = beta_2,
                   gamma = gamma,
                   delta = delta)) %>%
    df_deSolve_result() %>%
    mutate(alpha = alpha,
                   beta_1 = beta_1,
                   beta_2 = beta_2,
                   gamma = gamma,
                   delta = delta)
}
```

```
# Initiate dataset for plots and 
df_plot <- data.frame()

# Loop over aver all parameter values
for(alpha in c(0,0.1,0.2)) {
  for(beta_2 in c(0.7,1,1.5)) {
    # Add ODE solutions to dataset
    tmp <- make_df_sir_solution(beta_2 = beta_2, alpha = alpha)
    df_plot <- rbind(df_plot, tmp)
  }
}

# Plot the solutions as plots
(fig_4 <- df_plot %>%
  mutate(alpha_tex = factor(alpha, labels = c(TeX("$\\alpha=0$"),
                                              TeX("$\\alpha=0.1$"),
                                              TeX("$\\alpha=0.2$"))),
         beta_2_tex = factor(beta_2, labels = c(TeX("$\\beta_2=0.7$"),
                                                TeX("$\\beta_2=1.0$"),
                                                TeX("$\\beta_2=1.5$"))),
         name = factor(name, levels = c('S', 'I', 'R'))) %>%
  ggplot(aes(time, value, colour = name)) + 
  geom_line() + 
  labs(colour = 'Compartment', x = 'Time', y = '') +
  scale_x_continuous(breaks = c(0, 25, 50), expand = c(0, 0)) +
  scale_y_continuous(limits = c(0, 1), 
                     breaks = c(0, 0.5, 1), 
                     labels = c(0, 0.5, 1), 
                     expand = c(0, 0)) +
  facet_grid(vars(alpha_tex), vars(beta_2_tex), labeller = "label_parsed") +
  theme(panel.spacing = unit(0.5, "cm"))  +
  scale_color_manual(values = cbPalette[c(6, 5, 4)]))
```

```
# Function that returns steady state for given parameter values
i_steady <- function(alpha, beta_1 = 0.7, beta_2, gamma = 0.1, delta = 0.1) {
 tmp <- - (delta*(alpha*beta_2-beta_1*beta_2+gamma)) /
   (beta_1*beta_2*(alpha*beta_2+gamma+delta))
 # negative steady states are not meaningful
 if_else(tmp > 0, tmp, 0)
}

# Define all parameter configurations
all_combinations_steady <- expand.grid(alpha = seq(0,0.2,0.05),
                                       beta_2 = exp(seq(log(0.1),log(10),length.out=100)))

# Calculate the steady states for all considered configurations  
all_combinations_steady$infections <- mapply(FUN = i_steady,
                                             alpha = all_combinations_steady$alpha,
                                             beta_2 = all_combinations_steady$beta_2)

# Calculate the location of the steady state
get_beta_bar <- function(alpha = 0.2, beta_1 = 0.7, gamma = 0.1, delta = 0.1) {
  (alpha*gamma + sqrt(alpha^2*gamma*delta + alpha*beta_1*gamma^2 +
                        alpha*beta_1*gamma*delta)) /
    (alpha*beta_1-alpha^2)
}

# Define all parameter configurations
all_combinations_bar <- expand.grid(alpha = seq(0,0.2,0.05),
                                beta_1 = seq(0.5, 2, 0.01))

# Calculate the steady state positions for all considered configurations  
all_combinations_bar$beta_bar <- mapply(FUN = get_beta_bar,
                                        alpha = all_combinations_bar$alpha,
                                        beta_1 = all_combinations_bar$beta_1)
```

```
# Plot the steady states for the parameter configurations
p1 <- all_combinations_steady %>%
  ggplot(aes(x = beta_2, y = infections, colour = factor(alpha))) +
  geom_line() + xlab(TeX("$\\beta_2$")) + ylab(TeX("$I^*$")) +
  scale_x_log10(limits = c(0.1,NA), expand = c(0,0), labels = c(0.1, 1, 10)) +
  scale_y_continuous(expand = c(0.005,0.005), breaks = c(0, 0.2, 0.4)) +
  guides(col = guide_legend(title = TeX("$\\alpha$"))) +
  ggtitle("a)") +
  theme(plot.title = element_text(hjust = -0.2),
        legend.position="none") +
  scale_color_manual(values = cbPalette)

# Plot the steady state position of beta_2 for given beta_1
p2 <- all_combinations_bar %>%
  ggplot(aes(x = beta_1, y = beta_bar, colour = factor(alpha))) +
  geom_line() + xlab(TeX("$\\beta_1$")) + ylab(TeX("$\\beta_2^*$")) +
  ggtitle("b)") +
  theme(plot.title = element_text(hjust = -0.2)) +
  scale_y_continuous(limits = c(0, NA), expand = c(0.005,0.005), breaks = c(0, 0.5, 1)) +
  scale_x_continuous(expand = c(0,0)) +
  guides(col = guide_legend(title = TeX("$\\alpha$"))) +
  scale_color_manual(values = cbPalette)

(fig_5 <- p1 + p2)
```

```
set.seed(2)

# Utility function to generate sequence that is equidistant on log-scale:
log_seq <- function(from, to, length.out) {
  exp(seq(from = log(from), to = log(to), length.out = length.out))
}

# Define parameters that are constant for all simulation scenarios globally 
# instead of using them as parameters to simulate_abm
# to make them available later on 
# -> best compromise between style and simplicity:
N <- 1000 # total number of individuals

# The individuals are distributed over 
# - test individuals (beta distributed equidistant on log scale)
# - other individuals (beta drawn from log normals distribution)
nr_of_test_individuals <- 50

beta <- c(log_seq(0.001, 0.5, nr_of_test_individuals), 
          rlnorm(N - nr_of_test_individuals, meanlog = -4, sdlog = 1))

 # Number of time steps for simulation:
t_max <- 1e4
 # Number of infected individuals at start of simulation:
I_initial <- 10


# Definition of core simulation routine for model:
simulate_ibm <- function(
    a, d, c, scenario_name) {
  # Initially no previous immunization 
  # -> time since last immunization (denoted theta) = approx. Inf:
  theta <- rep(1e99, N) 
  nr_of_immunizations <- rep(0, N)
  time_since_last_transition <- rep(0, N)
  
  # Create variables that are used to record all state transitions along 
  # with the residence time and the beta value of the transiting individual:
  ii_residence_times <- 0
  residence_times <- rep(NA, N * t_max)
  compartment <- rep(NA, N * t_max)
  beta_transition <- rep(NA, N * t_max)
  
  # Matrix to store all state memberships throughout the simulation:
  states <- matrix(nrow = t_max + 1, ncol = N, 
                   dimnames = list(time = 0:t_max, beta = beta))
  # Initialize states at t = 0 (TRUE -> state = I, FALSE -> state = S):
  states[1, ] <- 1:N %in% sample(N, I_initial)
  
  # Main simulation loop
  # for all timesteps (index i), for all individuals (index j):
  for(i in 2:(t_max + 1)) {
    states[i, ] <- states[i - 1, ]
    I_div_N <- sum(states[i, ]) / N
    theta <- theta + 1
    time_since_last_transition <- time_since_last_transition + 1
    for(j in 1:N) {
      transition_from_compartment <- 'none'
      if(states[i, j]) { 
        # individual is in I:
        if(runif(1) < transition_prob_I_to_S(theta[j], a = a, d = d, c = c)) {
          # change to S:
          states[i, j] <- FALSE
          nr_of_immunizations[j] <- nr_of_immunizations[j] + 1
          theta[j] <- 0
          transition_from_compartment <- 'I'
        } 
      } else {
        # individual is in S:
        if(runif(1) < beta[j] * I_div_N) {
          # change to I:
          states[i, j] <- TRUE
          transition_from_compartment <- 'S'
        }
      } 
      # at end of simulation add artificial transition if appropriate:
      if(i == t_max + 1 & transition_from_compartment == 'none') {
        transition_from_compartment <- ifelse(states[i, j], 'I', 'S')
        if(transition_from_compartment == 'I') {
           nr_of_immunizations[j] <- nr_of_immunizations[j] + 1
        }
      }
      # record time in compartment if transition has occurred:
      if(transition_from_compartment != 'none') {
        ii_residence_times <- ii_residence_times + 1
        residence_times[ii_residence_times] <- time_since_last_transition[j]
        compartment[ii_residence_times] <- transition_from_compartment
        beta_transition[ii_residence_times] <- beta[j]
        time_since_last_transition[j] <- 0
      }
    }
  }
  
  # construct tibbles containing the results of the simulation:
  dat_sum <- tibble(beta = beta,
                    nr_of_immunizations = nr_of_immunizations,
                    burden = colMeans(states), 
                    scenario_name = scenario_name)
  
  states_long <- pivot_longer(as_tibble(states[ , 1:nr_of_test_individuals]), 
                         names_to = 'beta', 
                         values_to = 'state',
                         cols = everything()) |> 
    mutate(beta = as.numeric(beta),
           state = factor(ifelse(state, 'I', 'S'), levels = c('S', 'I')),
           time = rep(0:t_max, each = nr_of_test_individuals),
           scenario_name = scenario_name)
  
  fraction_infected <- tibble(fraction = rowMeans(states),
                              time = 0:t_max,
                              scenario_name = scenario_name)
  
  residence_times <- tibble(residence_times = residence_times, 
                            compartment = compartment,
                            beta = beta_transition,
                            scenario_name = scenario_name) |> 
    slice(1:ii_residence_times)
  
  # return results:
  return(list(dat_sum = dat_sum,
              states_long = states_long,
              fraction_infected = fraction_infected,
              residence_times = residence_times))
}

# Perform simulations:
scenario_names <- 
  fct_inorder(c('No specific immunity', 
                'Medium specific immunity', 
                'Strong specific immunity'))

none <- simulate_ibm(a = scenario_params$a[1], 
                     d = scenario_params$d[1], 
                     c = scenario_params$c[1], 
                     scenario_name = scenario_names[1])
medium <- simulate_ibm(a = scenario_params$a[2], 
                       d = scenario_params$d[2], 
                       c = scenario_params$c[2], 
                       scenario_name = scenario_names[2])
strong <- simulate_ibm(a = scenario_params$a[3], 
                       d = scenario_params$d[3], 
                       c = scenario_params$c[3], 
                       scenario_name = scenario_names[3])

# Collect simulation results from the different scenarios:
dat_sum <- 
  bind_rows(strong$dat_sum, 
            medium$dat_sum, 
            none$dat_sum)

states_long <- 
  bind_rows(strong$states_long, 
            medium$states_long, 
            none$states_long)

fraction_infected <- 
  bind_rows(strong$fraction_infected, 
            medium$fraction_infected, 
            none$fraction_infected)

residence_times <- 
  bind_rows(strong$residence_times, 
            medium$residence_times, 
            none$residence_times)
```

```
(fig_6 <- fraction_infected |> 
  ggplot(aes(time, fraction, colour = scenario_name)) + 
  geom_line(alpha = 0.3) + geom_smooth(se = FALSE) +
  labs(x = 'Time', 
       y = 'Fraction of infected individuals', 
       colour = 'Scenario') +
  theme(legend.position = 'right') +
  guides(color = guide_legend(nrow = 5, byrow = TRUE, title.position = 'top')) +
  scale_x_continuous(expand = c(0,0)) +
  scale_y_continuous(expand = c(0,0), limits = c(0, 0.5)) +
  scale_color_manual(values = cbPalette))
```

```
(fig_7 <- residence_times |> 
  mutate(Compartment = factor(compartment, levels = c('S', 'I'))) |> 
  group_by(beta, Compartment, scenario_name) |> 
  summarize(mean_residence = mean(residence_times)) |> 
  ggplot(aes(beta, mean_residence, colour = scenario_name)) + 
  geom_point(alpha = 0.2) + 
  facet_wrap(vars(Compartment), 
             scales = 'free', 
             labeller = label_both) + 
  scale_x_continuous(trans = 'log10', expand = c(0,0)) +
  scale_y_continuous(trans = 'log10', expand = c(0,0)) +
  labs(x = expression(paste('Infection rate', beta[i], ' (log scale)')), 
       y = 'Mean residence time (log scale)', 
       colour = '') +
  geom_smooth(se = FALSE) +
  theme(legend.position = 'bottom') +
  scale_color_manual(values = cbPalette))
```

```
# Calculate the disease burden
disease_burden <- dat_sum |> 
  mutate(burden = (burden * 1000) + t_max - 1000)

(fig_8 <- states_long |>  
  filter(time > t_max - 1000) |> 
  ggplot(aes(beta, time, fill = state)) +
  geom_tile() + 
  labs(x = expression(paste(beta[i], ' (log scale)')), 
       fill = 'Individual in compartment:') + 
  scale_colour_manual(values = c(gray(0.95), gray(0.7)), 
                      aesthetics = c('fill')) +
  geom_point(data = disease_burden, 
             alpha = 0.3,
             mapping = aes(beta, burden, 
                           fill = NULL, colour = scenario_name), 
             show.legend = FALSE, inherit.aes = FALSE) +
  geom_smooth(data = disease_burden, 
              se = FALSE,
              linewidth = 1,
              mapping = aes(beta, burden), 
              color = 'black',
              show.legend = FALSE, inherit.aes = FALSE) +
  scale_x_continuous(trans = 'log10',
                     expand = expansion(c(0, 0)),
                     breaks = c(0.001, 0.01, 0.1),
                     labels = c(0.001, 0.01, 0.1)) +
  scale_y_continuous(
    name = 'Time', 
    expand = expansion(c(0, 0)),
    sec.axis = sec_axis(~ (. - (t_max - 1000)) / 1000,
                        name = 'Fraction of time in I (coloured dots)')) +
  scale_color_manual(values = cbPalette) +
  facet_wrap(~ scenario_name, ncol = 3)  +
  theme(legend.position = 'bottom') + 
  guides(fill = guide_legend(ncol = 2, title.position = 'top')))
```
