# Supplementary material for: Impact of exposure frequency on disease burden of the common cold – A mathematical modeling perspective
Source: PLoS One. 2025 Oct 22;20(10):e0334527. doi: 10.1371/journal.pone.0334527 (PMC12543168; doi:10.1371/journal.pone.0334527)
Supplement: Attachment — Submitted filename: PLOS_D-24-38387-Review.pdf [file pone.0334527.s003.pdf]

The manuscript entitled "*Impact of exposure frequency on disease burden of the common cold - a mathematical modeling perspective*" investigates the impact of exposure frequency on infectious disease burden. The central argument, as I understand it, is that increased exposure boosts immunity via a specific immune response, and that beyond a certain threshold this heightened immunity may outcompete infection rates and ultimately lower disease burden. To study this hypothesis, the authors use two different modeling approaches:

1. an ODE-based (deterministic) SIRS model that includes a contact-frequency-dependent recovery rate; and
2. an individual-based (stochastic) SIS model in which the recovery rate wanes over time since an individual's last infection.

While the idea is interesting, I have concerns that the chosen model configurations may be either inappropriate or too vague to support the authors' conclusions.

In the ODE model, the transmission rate is first decomposed into a contact rate component (β_2) and an infection probability per contact component (β_1). The recovery rate is then augmented by a new term that is assumed to be boosted by the specific immune response in direct proportion to the contact rate β_2. As stated above, the assumption is that greater contact leads to more infections, which leads to heightened immunity, which leads to more rapid recovery. However, it is not clear to me why β_2 has been specifically isolated to investigate this effect. Would the same argument not equally apply to the transmission rate component β_1, the probability of infection per contact? In general, immunity should be boosted with increased infection - independent of whether infection was boosted as a result of increased contact (β_2) or infection probability (β_1). I understand that the focus of the investigation was the impact of exposure rates, but I am concerned that β_1 and β_2 are not so easily separated, and that the boosted recovery rate should depend on both quantities.

With respect to the individual-based SIS model, the principal issue is the seeming arbitrariness in how it and its parameters are configured. The manuscript mentions "test" subjects with transmission rates distinct from those of the other individuals, yet the motivation for these separate rates remains unclear. I am also unsure about the rationale behind the chosen parameter ranges for the test subjects, the distribution used for the remaining population, and the specific values of a, d, and c parameters across the "no," "medium," and "strong" model configurations. Even acknowledging that this model is not tailored to any specific pathogen (though the common cold is suggested as a possible example), the lack of a clear explanation or justification for these parameter choices makes it difficult to interpret the results. The only apparent rationale is to align the three residence time curves shown in the left panel of Figure 7.

In summary, while the premise of the study is intriguing, the current model implementations suffer from ambiguity in their parameter choices and their selective focus on contact rate as the driver of increased immunity. Because these issues cast doubt on the authors' conclusions, I cannot recommend this paper for publication in its current form. The models, as presented, appear either insufficiently defined or too vague to be of practical use.

I encourage the authors to consider revising both models to better justify the assumptions made and to clarify parameter selection and modelling decisions. Doing so could strengthen the manuscript and make its conclusions more convincing.

In addition to the general comments above, I also have the following minor comments:

1. The manuscript would benefit from additional proof-reading. There were many unresolved references and some erroneous text (see e.g., l119)
2. The paragraph on lines 44-50 could probably benefit from additional references – particularly the claim that "an increased number of sick days due to common colds was observed in the communities where the contact rates had been lower in the preceding years"
3. The sentence on l70: Do you mean SIS or SIRS model? For one, I don't believe the classical SIR model incorporates loss of immunity. Even in the SIRS model the loss of immunity is not instantaneous, it is exponentially-distributed.
4. L112: Even if the R compartment is not explicitly modelled, would it not still be an SIRS-like model is there is an immune state? Isn't infection, I, also an individual-based state?
5. I think the expression given for R_0 given between lines 235 and 236 is incorrect. First, the expression given suggests that this quantity would be negative if $\beta_1 < \alpha$, which is unphysical. Second, the model you propose is in effect still an SIRS model with a new recovery rate $\gamma^* = \gamma + \alpha\beta_2$ and a decomposed transmission rate $\beta = \beta_1\beta_2$. In this case, I think the reproduction number should be (assuming $N = 1$)
$$R_0 = \frac{\beta_1\beta_2}{\gamma + \alpha\beta_2}$$
6. In the expression for the endemic state there is in an error in bracket term of the numerator for R^*.
7. L268-269: Do you mean that individuals with large β_i values tend to have lower residence times (i.e., residence time and transmission rate are negatively correlated)?
