# Supplementary material for: Impact of exposure frequency on disease burden of the common cold – A mathematical modeling perspective
Source: PLoS One. 2025 Oct 22;20(10):e0334527. doi: 10.1371/journal.pone.0334527 (PMC12543168; doi:10.1371/journal.pone.0334527)
Supplement: Attachment — Submitted filename: response_to_reviewer_2.pdf [file pone.0334527.s004.pdf]

Response to reviewer 2

Reviewer 2: The manuscript entitled "Impact of exposure frequency on disease burden of the common cold - a mathematical modeling perspective" investigates the impact of exposure frequency on infectious disease burden. The central argument, as I understand it, is that increased exposure boosts immunity via a specific immune response, and that beyond a certain threshold this heightened immunity may outcompete infection rates and ultimately lower disease burden. To study this hypothesis, the authors use two different modeling approaches:
an ODE-based (deterministic) SIRS model that includes a contact-frequency-dependent recovery rate; and
an individual-based (stochastic) SIS model in which the recovery rate wanes over time since an individual's last infection.
While the idea is interesting, I have concerns that the chosen model configurations may be either inappropriate or too vague to support the authors' conclusions.

In the ODE model, the transmission rate is first decomposed into a contact rate component (β_2) and an infection probability per contact component (β_1). The recovery rate is then augmented by a new term that is assumed to be boosted by the specific immune response in direct proportion to the contact rate β_2. As stated above, the assumption is that greater contact leads to more infections, which leads to heightened immunity, which leads to more rapid recovery. However, it is not clear to me why β_2 has been specifically isolated to investigate this effect. Would the same argument not equally apply to the transmission rate component β_1, the probability of infection per contact? In general, immunity should be boosted with increased infection - independent of whether infection was boosted as a result of increased contact (β_2) or infection probability (β_1). I understand that the focus of the investigation was the impact of exposure rates, but I am concerned that β_1 and β_2 are not so easily separated, and that the boosted recovery rate should depend on both quantities.

Response: We agree that both components of the transmission rate—β_1 (infection probability per contact) and β_2 (contact rate)—contribute to overall infection risk and, by extension, to immune system stimulation.

In our model, we focused on β_2 as the driver of immune boosting because it captures the behavioral or environmental aspect of exposure: individuals who interact more frequently with others are more likely to be repeatedly exposed to pathogens. In contrast, β_1 is typically interpreted as a biological property of the pathogen-host

interaction (e.g., susceptibility, virulence, mucosal barrier integrity), which was not varied in our study.

That said, we agree that it would be both reasonable and biologically plausible to include β_1 as an additional or alternative driver of immune boosting—especially in models tailored to specific pathogens or datasets. For instance, the immune-stimulating term $(\gamma + \alpha\beta_2)*I$ could be extended to include a β_1-dependent component, such as $(\gamma + \alpha_1\beta_1 + \alpha_2\beta_2)*I$, in future versions of the model.

Our current model represents a simplified conceptual structure designed to isolate the effect of varying exposure frequency under minimal assumptions. We chose to vary β_2 in this first step to maintain transparency and interpretability. We now clarify this rationale in the discussion section of the manuscript and highlight the possibility of extending the immune feedback mechanism to include both β_1 and β_2 in future work (see lines 220 – 224 and lines 387 – 399 in file “article_diff.pdf”).

Reviewer 2: With respect to the individual-based SIS model, the principal issue is the seeming arbitrariness in how it and its parameters are configured. The manuscript mentions “test” subjects with transmission rates distinct from those of the other individuals, yet the motivation for these separate rates remains unclear. I am also unsure about the rationale behind the chosen parameter ranges for the test subjects, the distribution used for the remaining population, and the specific values of a, d, and c parameters across the “no,” “medium,” and “strong” model configurations. Even acknowledging that this model is not tailored to any specific pathogen (though the common cold is suggested as a possible example), the lack of a clear explanation or justification for these parameter choices makes it difficult to interpret the results. The only apparent rationale is to align the three residence time curves shown in the left panel of Figure 7.

Response: We acknowledge that the parameter choices in our individual-based SIS model are not derived from pathogen-specific empirical data and thus may appear arbitrary. However, our goal was to quantitatively test a general, mechanistic model that connects exposure frequency and average disease burden using reasonable parameter values. This approach serves as a proof of the principle possibility, rather than a predictive framework.

The inclusion of "test" subjects with distinct transmission parameters serves to simulate individuals with systematically higher or lower exposure frequencies, allowing us to examine the differential effects of exposure under otherwise controlled conditions. While the exact values of the parameters (such as *a*, *d*, and *c*) in the “no,” “medium,” and “strong” immune memory configurations were chosen heuristically they were selected to

produce qualitatively distinct and interpretable regimes of immune response behavior and consequently indeed, as you state, different residence times in the infected compartment. Similar average residence times across the scenarios as shown in the left panel of Figure 7 S arise naturally from the model structure. Characteristic differences in mean residence times in the compartment I (recovery times) are a consequence of the differences in the immune profile between the scenarios.

To address the reviewer's concern, we have now added a paragraph in the methods section (see lines 257 – 260 in file "article_diff.pdf") that provides a more detailed justification of the parameter choices, including the reasoning behind the distributions used and the intended conceptual contrasts among the configurations. We also explicitly note the exploratory nature of this modeling approach and suggest that future work could refine these assumptions using data from specific pathogens.

Reviewer 2: In summary, while the premise of the study is intriguing, the current model implementations suffer from ambiguity in their parameter choices and their selective focus on contact rate as the driver of increased immunity. Because these issues cast doubt on the authors' conclusions, I cannot recommend this paper for publication in its current form. The models, as presented, appear either insufficiently defined or too vague to be of practical use.
I encourage the authors to consider revising both models to better justify the assumptions made and to clarify parameter selection and modelling decisions. Doing so could strengthen the manuscript and make its conclusions more convincing.

Response: We thank the reviewer for the helpful hints. We incorporated them and adjusted our manuscript accordingly.

In addition to the general comments above, I also have the following minor comments:
The manuscript would benefit from additional proof-reading. There were many unresolved references and some erroneous text (see e.g., l119)

Response: We sincerely regret the oversight in the initial submission. We have since carefully proofread the manuscript, resolved the unresolved references, and corrected the identified textual errors, including the one noted at line 119.

Reviewer 2: The paragraph on lines 44-50 could probably benefit from additional references – particularly the claim that "an increased number of sick days due to common colds was observed in the communities where the contact rates had been lower in the preceding years"

Response: We have added a suitable reference (see line 65 in file “article_diff.pdf”).

Reviewer 2: The sentence on l70: Do you mean SIS or SIRS model? For one, I don’t believe the classical SIR model incorporates loss of immunity. Even in the SIRS model the loss of immunity is not instantaneous, it is exponentially-distributed.

Response: Thanks for the hint – indeed, we wanted to refer to the SIRS model, since you are absolutely right that the SIR model does not incorporate loss of immunity (see line 87 in file “article_diff.pdf”).

Reviewer 2: L112: Even if the R compartment is not explicitly modelled, would it not still be an SIRS-like model if there is an immune state? Isn’t infection, I, also an individual-based state?

Response: Indeed, while our model does not include a discrete "R" compartment in the classical compartmental sense, we agree that it shares conceptual similarities with SIRS models. In particular, the notion of immunity is captured through a continuously varying, individual-specific state variable rather than a binary classification (susceptible vs. resistant). Individuals with high immunity levels are effectively close to resistant to infection, but they still reside within the broader "susceptible" category in terms of model structure.

Thus, we consider our model as a generalization of the SIRS framework, where immune protection is treated as a gradual and dynamically evolving trait. We have clarified this point in the manuscript (see lines 436 – 442 in file “article_diff.pdf”) to avoid confusion and better align our terminology with standard epidemiological modeling concepts.

Reviewer 2: I think the expression given for R_0 given between lines 235 and 236 is incorrect. First, the expression given suggests that this quantity would be negative if $\beta_1<\alpha$, which is unphysical. Second, the model you propose is in effect still an SIRS model with a new recovery rate $\gamma^*=\gamma+\alpha\beta_2$ and a decomposed transmission rate $\beta=\beta_1 \beta_2$. In this case, I think the reproduction number should be (assuming N=1) $R_0=(\beta_1 \beta_2)/(\gamma+\alpha\beta_2)$

Response: You are absolutely right – thanks for pointing out this mistake! We have fixed the mistake in the manuscript (see line 292 in file “article_diff.pdf”) .

Reviewer 2: In the expression for the endemic state there is in an error in bracket term of the numerator for R^*.

Response: Again: You are absolutely right – thanks for pointing out this mistake! We have fixed the mistake in the manuscript (see line 300 in file “article_diff.pdf”).

L268-269: Do you mean that individuals with large β_i values tend to have lower residence times (i.e., residence time and transmission rate are negatively correlated)?

Response: Absolutely – thanks a lot for catching this incorrectness. We have fixed this point.
