# Supplementary material for: Impact of exposure frequency on disease burden of the common cold – A mathematical modeling perspective
Source: PLoS One. 2025 Oct 22;20(10):e0334527. doi: 10.1371/journal.pone.0334527 (PMC12543168; doi:10.1371/journal.pone.0334527)
Supplement: Attachment — Submitted filename: reply_to_reviewer_2.pdf [file pone.0334527.s005.pdf]

Response to reviewer 2

Reviewer #2: I appreciate the authors efforts to provide considered and eloquent responses to each item raised; however, I am concerned that some issues remain. Primarily, it is still difficult to know the extent to which the generated results apply to common cold viruses. The main result of the paper seems to be captured by the statement "we can observe a decreasing mean disease burden with a larger overall contact rate after a certain threshold for most parameter configurations". However, it is unclear what fraction of these "parameter configurations" are realized in practice. The authors acknowledge that "the framework is intentionally pathogen agnostic" and, without informed parameters, I would argue that this characterizes the analyses themselves - despite the title and narrative repeatedly anchoring to common cold pathogens.

Response: We fully acknowledge that the current model, while inspired by the clinical context of common cold infections, is fundamentally theoretical and pathogen-agnostic. More generally, the model could readily be applied to other common infections, such as viral gastroenteritis. Our primary aim was not to assert that a threshold-dependent reduction in mean disease burden occurs in *most* parameter configurations, but rather to demonstrate that such an effect is possible within the model—i.e., it emerges under certain plausible configurations of parameters.

At this stage, we are not in a position to determine how frequently or under what real-world conditions this phenomenon might occur, as this would require currently lacking robust empirical data to thoroughly calibrate all the model parameters. Instead, our goal was to propose and discuss a potentially relevant mechanism that consistently explains empirical observations and which merits further quantitative studies. We hope this modeling insight can serve as a foundation for future studies aimed at quantifying this effect in specific epidemiological contexts.

We have added an additional paragraph discussing these aspects in the discussion section (ll475-485).

Reviewer #2: Perhaps the authors observation that "Although the common cold can be caused by a wide variety of viruses, the clinical manifestations - particularly the duration and characteristics of symptoms - often show substantial overlap" could provide some opportunity to constrain or contextualize their model parameters, and in turn their results.

Response: We appreciate the reviewer's suggestion and agree that the observed overlap in clinical manifestations of common cold pathogens could, in principle, help to constrain or contextualize model parameters. However, given the current level of generality in our model and the lack of robust, pathogen-agnostic epidemiological data on relevant parameters (such as reinfection rates, immunity duration, or exposure frequencies) we believe that applying such constraints at this stage would be premature. Our goal in this work was to explore general, qualitative dynamics rather than to produce calibrated, pathogen-specific predictions (ll475-485). We see parameter refinement based on empirical data as an important step for future, more targeted model applications.

Reviewer # 2: l87: Could the authors elaborate on the sense in which immunity loss is instantaneous? In the standard SIRS model the immune period is exponentially distributed

Response: We apologize for the lack of clarity in our original phrasing. In the standard SIRS model, as correctly noted, the duration of immunity follows an exponential distribution, and the transition from the immune (R) to the susceptible (S) compartment occurs as a stochastic event. What we intended to highlight was that immunity in such models is typically represented as a binary state, in which individuals are either fully immune or fully susceptible, with an abrupt transition between these states once immunity "expires."

In contrast, our intention was to model immunity as a gradual, continuously varying property that wanes over time rather than being lost instantaneously when the cells transit from one to another state. This continuous representation allows for intermediate levels of immunity and more nuanced dynamics, which may better reflect real-world

immunological processes, particularly in the context of repeated exposures to common pathogens. We have revised the manuscript to clarify this point (ll75-78).

Reviewer # 2: l196-197: The authors state "there is no evidence that the infectivity of the pathogen should also affect the recovery rate". This seems to be contradicted by l392-394 of the discussion: "Immune boosting is likely influenced not only by the frequency of contacts but also by the probability and intensity of actual infections"

Response: We agree that there is a tension between these two statements and appreciate the reviewer's careful reading. We have revised the wording to express our reasoning more clearly and consistently (l192, ll388-392). Specifically, in the context of our model, a higher infectivity (β_1) does not directly affect the recovery rate. However, by leading to more frequent infections, it can indirectly enhance the immunity level, which in turn may increase the recovery rate. We hope this clarification resolves the ambiguity.

Reviewer # 2: l242-243: Related to the previous comment, I disagree with the statement that infectivity is an inherent, non-changing property of a specific virus, and that modulations of \beta_i directly represent changes in the contact rate in the model. Is it not possible that individual variation in viral load and severity would yield modulations in \beta_i independent of contact rates?

Response: We agree that factors such as viral load and disease severity can influence an individual's infectivity. In our modeling approach, we made the simplifying assumption that infectivity is constant across infected individuals. As such, variations in the parameter β_i were interpreted solely as differences in contact rates.

We acknowledge that this is a simplification. Incorporating individual variation in infectivity would certainly be a valuable extension of the model. However, doing so would require the introduction of an additional parameter or distribution, which we chose to avoid at this stage in order to maintain the conceptual clarity and tractability of the model. We have now clarified this modeling assumption in the main text (see ll239-241) and added a note to the discussion section (see ll445-452).

Reviewer # 2: l278: Full stop missing after "Fig. 4"

Response: We have added the full stop.

Reviewer # 2: l297: Repeated "the"

Response: We have deleted the extra "the".

Reviewer # 2: l300: I still worry there is an error in the endemic solutions. I think that the extra (separate) \gamma term in the numerator for the R* state is unnecessary. The authors (and readers) may find it helpful to re-express these solutions in terms of the reproduction number and compare the results with standard SIRS model solutions.

Response: We have now corrected the expression for the endemic equilibrium and apologize for having overlooked this issue in the first round of revisions. In addition, we have included the endemic solution for the classical SIRS model (ll184-186) with waning immunity for comparison. Since our ODE formulation is based on a straightforward parameter substitution ($\gamma \rightarrow \gamma + \alpha\beta_2$ and $\beta \rightarrow \beta_1\beta_2$), the structure of the analytical results is preserved. The classical SIRS model emerges as a special case of our framework when $\alpha\beta_2 = 0$ and β is replaced by any chosen value of $\beta_1\beta_2$ (ll298-304).
